# Chimeric Antigen Receptor T-Cells: The Future Is Now

**DOI:** 10.3390/jcm8020207

**Published:** 2019-02-07

**Authors:** Wassim Mchayleh, Prabhjot Bedi, Rajesh Sehgal, Melhem Solh

**Affiliations:** 1 Department of Medicine, Northside Hospital Cancer Institute, Atlanta, GA 30342, USA; wassimmchayleh@hotmail.com; 2 Department of Medicine, University of Pittsburgh Center East, Monroeville, PA 15146, USA; bedips@upmc.edu (P.B.); sehgalr2@upmc.edu (R.S.); 3 Blood and Marrow Transplant, Acute Leukemia and Immunotherapy Program, Northside Hospital, Atlanta, GA 30342, USA

**Keywords:** Chimeric, cellular, immunotherapy, T cell

## Abstract

The immune system acting via cancer immune-surveillance is considered a potential target for improving outcomes among some malignancies. The ability to harness immune cells, engineer them and educate them to target cancer cells has changed the paradigm for treating non-Hodgkin’s lymphomas (NHL) and acute lymphoblastic leukemia (ALL). Chimeric antigen receptor (CAR) T-cell therapy has shown remarkable anti-tumor activity against refractory B cell malignancies. Ongoing research aims to expand the scope of this adoptive cell therapy, understanding mechanisms of resistance and reducing toxicity. In this review, we will discuss the current scope of CAR T-cell therapy and ongoing future applications.

## 1. Introduction

Chimeric antigen receptor T-cell therapy ushers in a new dawn in the age of cancer immunotherapy. The ability of genetic engineering to alter the genome and induce specificity of T-cells against cancer cells in the form of chimeric antigen receptor is a revolutionary step in the rapidly evolving cancer treatment. It is a form of adoptive cell transfer (ACT). Initial studies using these cells have been promising [1]. Like other cancer therapies it is not sans the challenges but for sure is poised to provide clinicians with another resource in their armamentarium presently consisting of but not limited to surgery, chemotherapy, radiation and immuno-oncology agents like antibodies, cytokines, oncolytic viruses, bispecific molecules and cellular therapies [2]. Recent U.S. Food and Drug Administration “FDA” approval of chimeric antigen receptor “CAR-T” cell therapy for relapsed refractory (R/R) acute lymphoblastic lymphoma and for B cell NHL (Diffuse large B cell lymphoma) is encouraging and underscores the need for continued engagement in developing this novel therapy.

## 2. Structure and Mechanism of Action

The inherent innate and acquired anti-tumor immunity exists in body but is not as effective to fully contain the target resulting tumor overpowering it. However, this concept became the basis of modern-day immuno-oncology including adoptive cell therapy (ACT) which involves collecting and using immune cells to treat cancer. CAR-T cell therapy is a form of adoptive cell therapy which has recently gained attention due to success in clinical trials and FDA approval [1,3,4,5]. Chimeric antigen receptor—T cells or CAR T-cells are genetically engineered T cells expressing CARs on their surface. CARs are modified surface receptors that graft specificity to T-cells against a pre-identified target antigen being expressed on tumor cells. CD19 CAR-T cells express CARs targeted against CD19 which is expressed on normal B-cells as well as B-cell leukemias and lymphomas.

The prototypical T cell receptor consists of antigen-specific α and β chains linked with CD3 complex (ε, δ, γ and ζ chains). It identifies the target antigen presented in association with major histocompatibility complex (MHC). Chimeric antigen receptor (CAR) alternatively has an extracellular domain consisting of a single-chain variable fragment (scFv) recognizing a specific tumor antigen and is joined to a transmembrane domain which in turn is linked to signaling unit CD3ζ and costimulatory units CD28 or 4-1BB. This complex constitutes the intracellular domain [6,7]. CAR T-cells are able to identify specific tumor antigen-independent of the MHC complex.

**Figure 1:**
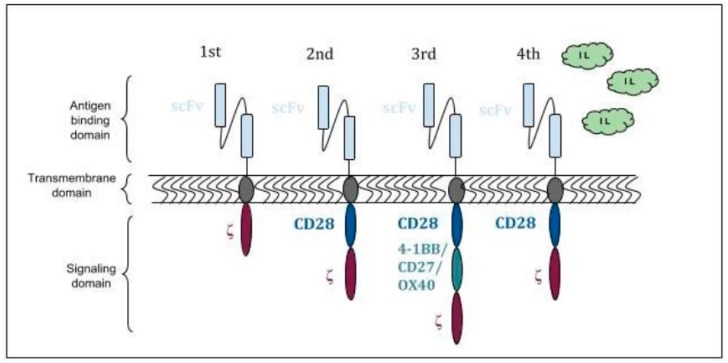
Chimeric antigen receptor (CAR) structure and generations.

The first generation of CARs contained only CD3ζ as a signaling molecule and even though the T cells expressing these CARs killed the target cells, they did not proliferate readily and failed clinical trials [8,9]. This barrier has been overcome by the development of second and third generation CARs (Figure 1). These have intracellular domain consisting of costimulatory molecules CD28 or 4-1BB linked in tandem with CD3ζ. The activating signal is provided by CD3ζ. The costimulatory molecules enhanced cytokine production, proliferation and survivability [10]. These have intracellular domain consisting of 1 or 2 additional costimulatory molecules such as CD28, 4-1BB, OX40 or CD27 linked in tandem with CD3ζ. The activating signal is provided by CD3ζ. The costimulatory molecules enhanced cytokine production, proliferation and survivability [9]. The fourth generation “Armored CAR T-cells” have now been developed in a bid to overcome inhibitory tumor microenvironment, especially in solid malignancies. Armored CARs capable of secreting cytokines as IL-2, IL-12 transcribed from an independent gene incorporated into and transduced by the CAR vector in addition to typical CAR functionality [11,12]. IL-12 secreting anti-MUC-16^ecto^ antigen CARs against ovarian cancer cells have demonstrated success in murine models [13].

CAR T-cell therapy involves extracting a patient′s normal T-cells via leukapheresis, selection, activation, transduction to express CARs using lentiviral or retroviral vectors, expansion of transduced cells and infusion of the final product back to the patient. After the CAR T-cells are infused back into the patient, the engineered cells proliferate, recognize and kill tumor cells bearing the specific antigen the CAR is directed against. Most of the current clinical trials have been with anti-CD19 CAR T-cells which are directed against CD19 antigen [14,15]. Lymphodepleting chemotherapy is administered prior to CAR T-cell. It results in endogenous cytokine production stimulating CAR T-cells expansion and proliferation [16,17]. 

As B-cells express molecules like CD19, CD20 and CD22 which are specific to their lineage and not expressed on other body cells, the tumors arising from them became the target of interest for development for CAR T-cells. CD19 was chosen as one of the first targets because of its greater expression relative to other antigens [6]. Anti CD19 CAR T-cells are also the most widely studied amongst others in current clinical trials.

## 3. FDA Approved Indications

There have been 3 recent US Food and Drug Administration (FDA) approvals for using anti CD19 CAR T-cells in B-Cell malignancies because of success demonstrated in clinical trials [1,4,5].

Tisagenlecleucel (KYMRIAH) was approved for refractory or relapsed B-cell precursor ALL in children and young adults <25 years old in August 2017 and relapsed or refractory (RR) diffuse large B-cell lymphoma (DLBCL) not otherwise specified, high-grade B-cell lymphoma and DLBCL arising from follicular lymphoma in May 2018 [18]. Tisagenlecleucel is CD19 directed 4-1BB/CD3ζ CAR T-cell therapy. It consists of autologous T-cells genetically modified to express anti-CD19 scFv linked to signaling domains 4-1BB and CD3ζ.

Axicabtagene ciloleucel (YESCARTA) is CD19 directed CD28/CD3ζ CAR T-cell therapy which was approved for relapsed or refractory (R/R) diffuse large B-cell lymphoma (DLBCL) not otherwise specified, primary mediastinal large B-cell lymphoma, high-grade B-cell lymphoma and DLBCL arising from follicular lymphoma in October 2017 [19].

## 4. Acute Lymphoblastic Leukemia (ALL)

Chemotherapy remains the first line of treatment for childhood and adult acute lymphoblastic leukemia (ALL). Because of an outstanding advancement in the treatment of childhood disease, the 5-year survival rate is approaching 90% in some countries [20]. On the other hand, the majority of adults with the disease will relapse after initial remission and a significant proportion (25%) will have resistant disease resulting in high overall mortality [21].

CD19 CAR T-cell therapy was studied in a number of single-center studies for R/R ALL which showed promising results as evidenced by achievement of complete remission (CR) in 70%–97% of patients [22,23,24,25,26]. The successful results of ELIANA (Determine Efficacy and Safety of CTL019 in Patients with Relapsed and Refractory B-cell ALL) multicenter trial using CD19 scFv/4-1BB/CD3ζ construct of CAR T-cells formed the basis of first FDA approval of CAR T-cell therapy. Out of 107 patients screened, 92 were enrolled and 75 received the CAR T-cell treatment. The median marrow blast percentage was 74% (range 5 to 99) and 61% of patients had undergone previous allogeneic hematopoietic stem-cell transplantation. The overall remission was attained in 81% with 60% attaining a complete remission and 21% achieving a complete remission without hematologic recovery. However, on an intention to treat analysis of the full enrolled population (*n* = 92), the overall remission rate was 66%. Among patients with complete remission, the relapse-free survival at 12 months was 59% (95% CI, 41 to 73) [1]. 

A single center report from Memorial Sloan Kettering Cancer Center showed that among 53 adults who received 19-28z CAR T cells, complete remission (CR) was obtained in 83% of the patients with a median event-free survival of 6.1 months and overall survival of 20.1 months. Survival rates were significantly better for patients with low disease burden. It is noteworthy in this study as in the ELIANA trial, 83 patients were enrolled in the study but only 53 patients received the infusion of CAR-T cells [27]. These studies show that although CAR T cell therapy is effective in ALL, it is limited by the ability to get patients to this treatment in a timely fashion given the highly proliferative nature of relapsed and refractory ALL as well as logistical issues encompassing CAR T-cell manufacturing and administration.

Tisagenlecleucel (KYMRIAH) is currently approved for refractory or relapsed B-cell precursor ALL in children and young adults of age <25. The current FDA approved indications are summarized in Table 1.

## 5. Non-Hodgkin′s Lymphoma (NHL)

As CD19 is also expressed in B-cell NHL′s, CD19 CAR T-cells have been studied in single and multicenter studies for the treatment of some forms of R/R B-cell NHL like follicular lymphoma, transformed follicular lymphoma, diffuse large B-cell lymphoma and primary mediastinal B-cell lymphoma with encouraging results [4,5,28].

As a result of success in these multicenter trials, Axicabtagene ciloleucel (YESCARTA) and Tisagenlecleucel (KYMRIAH) were approved by FDA for (R/R) diffuse large B-cell lymphoma (DLBCL) not otherwise specified, primary mediastinal large B-cell lymphoma, high-grade B-cell lymphoma, DLBCL arising from follicular lymphoma and (RR) diffuse large B-cell lymphoma (DLBCL) not otherwise specified, high-grade B-cell lymphoma and DLBCL arising from follicular lymphoma respectively. The trial involving Axicabtagene ciloleucel enrolled 111 patients out of which 101 received the treatment. Overall response rate (ORR) and complete remission (CR) criteria were met by 82% and 58% respectively at a median of 15.4 months follow-up [4]. The ZUMA-1 trial, a multicenter single-arm registration trial at 22 sites in the USA and Israel, 119 patients with histologically confirmed large B-cell lymphoma including DLBCL, mediastinal B-cell lymphoma and transformed follicular lymphoma were enrolled and 108 received Axicabtagene ciloleucel at a target dose of 2 × 10 (6) CAR T cells per KG after a lymphodepleting chemotherapy of fludarabine and cyclophosphamide. After a median follow up of 27.1 months, 101 assessable patients were included and 84 (83%) had an objective response and 59 (58%) had a complete response. The median duration of response, progression-free survival and overall survival of 11, 6 and >27 months, respectively. Similarly, the JULIET study assessed 93 patients with relapsed refractory DLBCL who received Tisagenlecleucel with a median follow up time of 14 months. The best overall response rate was 52% with 40% achieving a CR. At 12 months after the initial response, the rate of relapse-free survival was estimated to be 65% (79% among patients with a complete response) [5]. Both studies establish CAR T cell therapy to be an effective treatment for relapse refractory lymphoma with some patients achieving long sustainable responses.

## 6. Toxicity

The CAR T-cell like other cancer therapies is not free of undesirable effects. Even though this therapy provides a potential treatment modality where none existed, it has quite a few worrisome and potentially fatal toxicities.

Several mechanisms play a role in orchestrating the detrimental effects of CAR T-cell infusion. It might be an “on target” effect resulting from intense cytokine release from infused CAR T-cells or damage inflicted to normal tissue by CAR T-cells as a result of that tissue expressing either target antigen or a protein which cross-reacts with the CAR. Allergic reactions and Tumor lysis syndrome have also been reported [29,30,31].

The two frequently reported specific toxicities to include cytokine release syndrome (CRS) and neurotoxicity [32].

CRS is a common and concerning toxicity of CAR-T cell therapy. The onset is usually within the first week of infusion. After infusion, the T-cells undergo activation, proliferation and mount a vigorous cytokine response. The cytokines implicated in CRS are produced from CAR T-cells as well as the other immune cells activated by the cytokines released from the CAR T-cells. This results in an exuberant systemic inflammatory response manifesting as a noninfectious flulike illness. CRS can potentially affect all organ systems but more commonly characterized by fevers, hypotension, tachycardia, respiratory and renal insufficiency [33]. High tumor burden, a higher concentration of CD19 cells in the bone marrow, pretreatment thrombocytopenia and high CAR T-cell were found to be risk factors for CRS in a study of 133 patients with R/R CD19+ B-cell malignancies who received lymphodepleting chemotherapy followed by CD19 CAR T-cells [34]. CRS is characterized by high levels of cytokines like interleukin-6 (IL-6) and interferon-γ (IFN-γ). Tocilizumab (Actemra) is an IL-6 antagonist which is effective in treating CRS and has been approved by FDA [23,30]. Glucocorticoids are indicated if there is a lack of rapid response to Tocilizumab [6]. Supportive care should be provided in an ICU or floor setting depending on the grade of severity. 

Neurotoxicity has been reported by multiple research groups testing CD19 CAR T-cells [23,35]. Commonly reported symptoms to include headaches, delirium, aphasia, focal neurological deficits, seizures and loss of consciousness. The pathophysiology of neurological symptoms has not been elucidated yet but it is usually fully reversible barring few cases of fatal cerebral hemorrhage or edema [32,36].

B-cell aplasia is another frequently reported adverse effect [37]. It results from CD19 CAR T-cells targeting normal non-malignant CD19+ B-cells resulting in their elimination. This, in turn, leads to hypogammaglobinemia predisposing patients to opportunistic infections. Immunoglobulin levels are monitored after infusion of CAR T-cells and hypogammaglobinemia is treated with IVIG infusions. It is expected collateral damage of the therapy and B-cells recover as the number of CAR T-cells decline.

As is the case with other immunomodulation therapies, treatment with CAR T-cell can predispose to infections. Patients receiving CAR T-cells are frequently lymphopenia and neutropenia making them susceptible to opportunistic infections. There should be a low suspicion to suspect infection especially in patients who develop CRS as clinical features of both frequently overlap.

Because of the risk of serious toxicities, FDA approval of CAR T-cells was granted contingent with a Risk evaluation and mitigation strategy which requires the physicians and hospital staff where the drug is administered to be trained in the management of adverse effects.

## 7. Mechanisms of Resistance

Several mechanisms of development of resistance to CAR therapy have been described in the literature. These include anti-idiotype antibodies, tumor antigen escape, antigenic target gene mutation, insertion of CAR into leukemia cells [38,39,40,41].

The scFvs used for manufacturing CARs are sourced from murine monoclonal antibodies and hence are at risk for inducing production of anti-idiotype and anti-mouse antibodies which are inhibitory to CAR T-cell action rendering the therapy ineffective [38]. Single antigenic domain scFvs which have smaller ectodomain and hence lesser immunogenicity have been developed to overcome this limitation, however, the downside is weaker affinity and lesser specificity [42]. Another approach is to use human scFvs which will eliminate the formation of anti-mouse antibodies but nevertheless, the possibility of anti-idiotypic antibody response persists. The construct of Anti-HER2, EphA2 and mesothelin CARs reflect this approach [43,44,45].

Downregulation or loss of target antigen expression on the leukemia cells is a commonly described mechanism resulting in relapse [39]. The significant amount of data in this regard comes from the studies involving the use of anti-CD19 CARs in pediatric ALL patients. Antigen escape and lineage switch are two different means of CD19 loss described in the literature [46]. Whereas in case of antigen escape relapse after remission occurs with the phenotypically similar disease but CD19- cells, lineage switch results on phenotypically different but genetically related malignancy, for example, acute myeloid leukemia (AML) [39]. The immune pressure from CD19 CAR T-cells can result in the selection of exon 2 splice variants resulting in loss CD19 epitope expression [47]. However, further investigations into this have shown that anti-CD19 CARs, in fact, bind exon 4 and loss of CD19 expression in exon 2 variant results from deranged CD19 surface localization [48,49]. Designing CARs targeting more than one antigen can potentially aid in overcoming antigen escape.

The immune strain resulting from CD19 CAR T-cells has been speculated to induce post-therapy mutations resulting in lack of CD19 on mutated B-ALL tumor cells as evidenced by the work of Orlando et al. involving patients with CD19-negative disease relapse [50].

Most recently, there was a case report where a CAR gene was inadvertently incorporated into a leukemic B-cell during T-cell construction. This, in turn, resulted in a product which would bind to CD19 epitome on the surface of the leukemic cell and hence mask it from recognition by the therapeutic T-cells. The patient exhibited florid relapse as evidenced by >90% infiltration of marrow by CD10+CD19- leukemic cells as well as blasts in circulation. Further investigation revealed these were, in fact, CAR-transduced B-cells. The patient was treated with salvage chemotherapy and immunotherapy but eventually passed away [41].

## 8. Future indications and Solid Tumors

The backbone of CAR T-cell therapy is the identification of target antigen and the development of CAR T-cell construct against that antigen. An ideal antigen would be a one with higher and dense expression in the target tissue and not expressed on normal cells. This would augment the efficiency of the therapy and minimize collateral damage to normal tissue. This underlying concept forms the basis of the present success of CD19 directed therapy as CD19 is B-cell lineage specific antigen. B-cell aplasia noted as one of the adverse effects is a result of collateral damage by virtue of CD19 expression on normal B-cells.

A significant hurdle and challenge in extrapolating CAR T-cell to other hematological cancers and potentially solid cancers will be the identification of suitable target antigens. This is exemplified by the challenges encountered in the development of CAR T-cells against T cell malignancies as shared antigens make the differentiation between the malignant, normal and therapeutic T-cells onerous resulting in T-cell aplasia and mutual killing of CAR T-cells (fratricide) as unintended consequences [51]. Some other malignancies which are presently being investigated and have the potential for success in future are CLL, Multiple myelomas, Hodgkin lymphoma, anaplastic large-cell lymphoma, AML. Each one has their own specific challenges, however, the perennial and swift developments in genetic engineering have the potential to overcome them sooner or later.

Even though CAR T-cell therapy has shown encouraging results in some hematological cancers as mentioned above, its application to solid tumors is fraught with challenges including but not limited to identification of a precise tumor-specific antigen, immunosuppressive nature of tumor microenvironment [52], limited and poor persistence after infusion [53]. Multiple trials using CAR T-cells in solid malignancies have demonstrated severe toxicities as even minimal expression of target antigen in normal host tissues becomes a victim of the vigorous CAR T-cells directed against that antigen [54]. Some examples are: CAR T-cells directed against carbonic anhydrase IX (CAIX) in renal cell cancer resulted in hepatotoxicity due to the expression of CAIX on bile duct epithelium [55,56], ERBB2 CAR T-cells used in metastatic colorectal cancer caused pulmonary toxicity due to antigen expression on lung epithelium along with multi-organ failure and ultimate demise of patient. The trial using CEACAM5-CAR T-cells for gastrointestinal tumors was closed prematurely due to poor efficacy and toxicities [53]. Even though some tumor-specific antigens have been identified and trials involving CAR T-cells directed against these showed a better safety profile but clinical efficacy was limited. HER2-based CAR used for sarcoma is one such example which in trial displayed a favorable safety profile but treatment outcome no better than a stable disease [57]. One ray of light has been the success with glioblastoma. CAR T cells developed against IL-13Rα were used via local intracranial infusions for recurrent glioblastoma in one patient with intracranial and spinal tumors following which regression of all intracranial and spinal tumors was exhibited and the response continued for 7.5 months [58].

Despite the early success, this field is still in its stages of inception and it might be a while before it sees the light of the day in terms of establishing itself as conventional therapy. The length of time needed to get patients ready for infusion associated with the big price tag with therapy itself and an equally sizeable healthcare costs emanating from its administration are significant limiting factors to providing CAR T-cell therapy on a wide range. One of the areas of current research involving adoptive cell therapy is the development of allogeneic T-cells that can serve as universal off the shelf CAR T-cells facilitating more widespread clinical use. This has been made feasible by genome editing techniques like ZFN (zinc finger nuclease), TALEN (transcription activator-like effector nuclease) and CRISPR-Cas9 [59]. UCART19 is one such example. It has been developed with lentiviral transduction and as well as TALEN mediated gene editing of T cell receptor α chain and CD52 gene loci. Successful molecular remission was demonstrated in two infants with relapsed refractory CD19+ B cell ALL with a single dose of UCART19 cells infused after administering lymphodepleting chemotherapy and anti-CD52 serotherapy. The investigators in this trial used this strategy as a bridge to stem cell transplantation [60]. FT819 is a human induced pluripotent stem cell-derived CAR-T cell product which exhibited an efficient cytotoxic response in pre-clinical in vitro studies [61]. Further research into faster manufacturing and cell expansion, utilizing off the shelf ready products with lower toxicity will help make CAR T-cell therapy more available and affordable.

## Figures and Tables

**Table 1:** Approved U.S. Food and Drug Administration (FDA) Indications for Chimeric antigen receptor T-cell (CAR T-Cell) Therapy.

CAR T-Cell Product	CAR Construct	FDA Approved Indications
Tisagenlecleucel (KYMRIAH)	CD19scFv/4-1BB/CD3ζ	• B-Cell acute lymphoblastic leukemia (ALL) that is refractory or in the second relapse in patients up to age 25 years [18] • Adult patients with (r/r) large B-Cell lymphoma after two or more lines of systemic therapy including diffuse large B-cell lymphoma (DLBCL) not otherwise specified, high-grade B-cell lymphoma and DLBCL arising from follicular lymphoma [18].
Axicabtagene ciloleucel (YESCARTA)	CD19scFv/CD28/CD3ζ	• Adult patients with (r/r) large B-cell lymphoma after two or more lines of systemic therapy, DLBCL not otherwise specified, primary mediastinal large B-cell lymphoma, high-grade B-cell lymphoma and DLBCL arising from follicular lymphoma [19].
